# RE: Long-term efficacy of microbiology-driven periodontal laser-assisted therapy, by Martelli et al., *Eur J Clin Microbiol Infect Dis* 2016, 35(3): 423-431

**DOI:** 10.1007/s10096-016-2785-7

**Published:** 2016-10-13

**Authors:** C. Gatti, M. Aimetti, G. Rasperini, L. Landi, F. Cairo, on behalf of SIdP, Italian Society of Periodontology and Implantology

**Affiliations:** 1 Viale dei Mille, 9, 50131 Firenze, Italy; 2 via fra’ G. Angelico 51, 50121 Firenze, Italy

Dear Editor,

We read with interest the manuscript by Dr Martelli et al. entitled “*Long-term efficacy of microbiology-driven periodontal laser-assisted therapy*” [1], recently published in the *European Journal of Clinical Microbiology & Infectious Diseases* (2016 Mar; 35(3): 423–431). In this manuscript, the authors described the treatment of periodontal disease in more than 2,000 patients, and reported clinical and microbiological findings after 2 years. As board members of the Italian Society of Periodontology and Implantology (SIdP) we would like to draw attention to some critical points that may affect the study outcomes and conclusions.

## Diagnosis

The diagnosis of periodontal disease as applied by the authors is far from the current international standard [2]. In the present manuscript, information with regard to mean clinical attachment level, full-mouth plaque score, full-mouth bleeding score and tooth loss is unknown. In addition, the diagnosis of severe periodontal disease applies when more than 30 % of sites are involved in chronic periodontitis, while for aggressive periodontitis this definition should be extended with the involvement of at least three additional teeth to molars/incisors. The definition of disease severity used by the authors is based on personal criteria. Furthermore, information with regard to possible risk factors for periodontal disease is limited to smoking habits only, although the detrimental effect of other risk factors or potential risk factors (such as diabetes, systemic diseases, immunodeficiency, drugs, osteoporosis, stress, obesity, etc.) in terms of disease progression and treatment outcomes is very well known [3].

## Treatment

In the additional material section, the authors stated “*An accurate mechanical ultrasound for SRP was performed according to the needs of the patients and up to complete satisfaction of the clinicians. After completion of SRP, patients were treated in all pockets with a 1064 nm Nd:YAG laser*” [1]. This means that classical mechanical sub-gingival debridement of root surfaces (SRP) was performed at each experimental site before laser application and the final delivered therapy is a combination of SRP and laser treatment. Since the study is a case series lacking a control group (single treatment with SRP or laser) it is impossible for the reader to assess if final benefits are associated or not with laser application. A randomized clinical trial (RCT) is mandatory to investigate the efficacy of combined therapy versus single treatments, and to understand the potential additional benefits of laser application. In the last 15 years, RCTs from several independent universities and systematic reviews (SR) clearly showed a similar efficacy in applying classical therapy (mechanical SRP) or laser, and no additional benefits in adding laser to mechanical treatment [4–7]. These findings suggest that caution must be taken with laser application for periodontal treatment, also considering the cost–benefit ratio [4–7]. In fact, as demonstrated approximately 15 years ago [8, 9], similar clinical and microbiological outcomes to those reported by Martelli et al. [1] may be easily achieved using conventional therapy, with significantly less costs. The reader should also keep in mind the long-term clinical efficacy of traditional periodontal therapy, which is supported by several studies with more than 10 years of follow-up [10, 11].

Finally, in the present study [1] the full-mouth treatment, meaning the treatment of all involved sites in the patient mouth, is not clearly defined, and this is another critical point, since patients were described by the authors as affected by “advanced” chronic or aggressive periodontitis.

## Follow-up

The authors defined as a “long-term” observation the final 2-year follow-up. This is another surprising aspect of this manuscript, since in periodontal literature the long-term effect of therapy is usually defined after at least 5 years of observation [12]. Conversely, the manuscript lacks critical information for the reader, including tooth loss (teeth extracted for advanced bone destruction) at final follow-up. The latter reflects the obvious primary treatment goal and the type of re-treatment of sites showing progression of periodontitis. Statistical analysis alone is not able to provide information related to prediction of disease progression and tooth loss at final follow-up. Furthermore, information regarding the secondary prevention of enrolled patients is unknown, even if authors generically reported the additional use of laser “*only by teeth showing periodontal pockets with PPD>2 mm*”.

## Statistical analysis

Surprisingly, the authors used a *t*-test for statistical analysis, which is not adequate in this type of study since multilevel analysis [13, 14] is generally used to assess the effect of prognostic factors on treatment outcomes. This approach allows the analysis of a multifaceted scenario that is non-surgical treatment of periodontal disease, where every variable is interconnected and able to influence each other at different levels.

## Ethics

Although the authors reported that “*there are no experiments requiring appropriate institutional review board(s) approval*”, it is surprising that no ethical board approval was reported, since the applied procedure is not a standard technique for the treatment of periodontal disease, and the clinical benefits are questionable.

Based on our criticisms, the manuscript conclusion “*This study, conducted for the first time on such a large series, clearly demonstrates long-term efficacy of microbiology-driven non-invasive treatment of periodontal disease*” is, in our opinion, erroneous since not supported by the applied methodology during the study. We do not believe that the present study provides useful clinical information in periodontal therapy.

Best regards

The board of the SIdP, Italian Society of Periodontology and Implantology

Claudio Gatti, *President*

Mario Aimetti *Elected President*

Giulio Rasperini *Vice President*

Luca Landi *Treasurer*

Francesco Cairo *General Secretary*
